# Influence of temperature fluctuations during cryopreservation on vital parameters, differentiation potential, and transgene expression of placental multipotent stromal cells

**DOI:** 10.1186/s13287-017-0512-7

**Published:** 2017-03-11

**Authors:** Denys Pogozhykh, Olena Pogozhykh, Volodymyr Prokopyuk, Larisa Kuleshova, Anatoliy Goltsev, Rainer Blasczyk, Thomas Mueller

**Affiliations:** 1 Institute for Transfusion Medicine, Hannover Medical School, Carl-Neuberg-Straße 1, 30625 Hannover, Germany; 2 Institute for Problems of Cryobiology and Cryomedicine, National Academy of Sciences of Ukraine, Pereyaslavskaya Str. 23, 61015 Kharkiv, Ukraine; 3 Synlab Medical Care Center Weiden Ltd., Zur Kesselschmiede 4, 92637 Weiden in der Oberpfalz, Germany

**Keywords:** Placenta, Amnion, Cryopreservation, MSC, Biobanking, Cryomicroscopy, Viability, Mimicking long-term storage, Temperature fluctuations

## Abstract

**Background:**

Successful implementation of rapidly advancing regenerative medicine approaches has led to high demand for readily available cellular suspensions. In particular, multipotent stromal cells (MSCs) of placental origin have shown therapeutic efficiency in the treatment of numerous pathologies of varied etiology. Up to now, cryopreservation is the only effective way to preserve the viability and unique properties of such cells in the long term. However, practical biobanking is often associated with repeated temperature fluctuations or interruption of a cold chain due to various technical, transportation, and stocking events. While biochemical processes are expected to be suspended during cryopreservation, such temperature fluctuations may lead to accumulation of stress as well as to periodic release of water fractions in the samples, possibly leading to damage during long-term storage.

**Methods:**

In this study, we performed a comprehensive analysis of changes in cell survival, vital parameters, and differentiation potential, as well as transgene expression of placental MSCs after temperature fluctuations within the liquid nitrogen steam storage, mimicking long-term preservation in practical biobanking, transportation, and temporal storage.

**Results:**

It was shown that viability and metabolic parameters of placental MSCs did not significantly differ after temperature fluctuations in the range from –196 °C to –100 °C in less than 20 cycles in comparison to constant temperature storage. However, increasing the temperature range to –80 °C as well as increasing the number of cycles leads to significant lowering of these parameters after thawing. The number of apoptotic changes increases depending on the number of cycles of temperature fluctuations. Besides, adhesive properties of the cells after thawing are significantly compromised in the samples subjected to temperature fluctuations during storage. Differentiation potential of placental MSCs was not compromised after cryopreservation with constant end temperatures or with temperature fluctuations. However, regulation of various genes after cryopreservation procedures significantly varies. Interestingly, transgene expression was not compromised in any of the studied samples.

**Conclusions:**

Alterations in structural and functional parameters of placental MSCs after long-term preservation should be considered in practical biobanking due to potential temperature fluctuations in samples. At the same time, differentiation potential and transgene expression are not compromised during studied storage conditions, while variation in gene regulation is observed.

**Electronic supplementary material:**

The online version of this article (doi:10.1186/s13287-017-0512-7) contains supplementary material, which is available to authorized users.

## Background

Recent advances in cellular therapy, regenerative medicine, and stem cell research have substantially increased the need for various types of readily available cellular suspensions, but in particular the need for multipotent stromal cells (MSCs). Numerous clinical trials have proved the efficiency of the application of MSCs in the treatment of various severe pathologies, wound healing, and regulation of the endocrine system [1–3]. In addition, progress in the development of biocompatible materials opens great potential for the application of MSCs in tissue engineering due to the high differentiation potential and proliferation capacity of these cells [4, 5].

While numerous organs and tissues can serve as a source of MSCs, various components of placenta are among the most prolific for application in regenerative medicine [6–8]. MSCs of placental origin can be derived from placental villi, placental membranes, umbilical cord cells, and Wharton’s jelly [9–11]. Among the major advantages of placenta as a source of MSCs are the opportunity to recover a large amount of initial material, the possibility of obtaining autologous material without invasive surgery, and the relative simplicity of retrieving high amounts of the cells [12]. Furthermore, the developmental origin of placental tissues, being partially maternal and partially fetal [13], allows us to receive two different patient-specific materials at the same time, one for the fetus and the other for the mother.

Development of storage and transportation protocols is the crucial condition for the clinical application of placental MSCs. Presently, application of low-temperature storage technologies is the only effective approach for the long-term preservation of various cell suspensions and other biomaterials [14, 15]. In order to be clinically sufficient, such technologies should not only provide a high viability of the preserved material, but also comply with modern Good Manufacturing Practice (GMP) standards [16, 17]. In our previous work we discussed the importance and demonstrated possibilities for the utilization of low-temperature storage technologies with materials and equipment approved for medical practice [18]. Furthermore, we have demonstrated generation and cryopreservation of alginate scaffolds with placental MSCs in an animal model [19, 20].

Temperature regime is one of the key components of the cryopreservation procedure [21]. For the purposes of long-term preservation it is generally accepted to freeze suspensions of MSCs at the rate of 1 °C/min down to –80 °C with immersion into liquid nitrogen [22]. Long-term storage of biological material in liquid nitrogen is by far the most practical and reliable method since the temperature of liquid nitrogen is constant. At the same time, practical functioning of low-temperature banks often requires frequent access to material and may be accompanied by various technical, stocktaking, and other events that may be associated with temperature fluctuations due to changes in the location of storage of biological objects, their transportation, and similar alterations. Therefore, even with modern biobanking technology, repeated temperature fluctuations or interruption of a cold chain may occur during maintenance and handling.

During cryopreservation, biochemical and cellular processes are anticipated to be on hold for a virtually infinite time. However, fluctuations of the temperature may lead to accumulation of stress in samples [23] or to periodic release of unfrozen bound water fractions [24], possibly compromising survival and vital parameters of cells. Therefore, such fluctuations could be a main factor in potential damage during long-term storage of cryopreserved biomaterial. At the same time, there is a lack of information and studies which focus on the analysis of this problem.

In addition to viability and overall structure-functional parameters, cryopreservation can also influence certain other cellular pathways and molecular mechanisms [25]. Wide application of modern molecular biology methods in stem cell research, e.g., generation of induced pluripotent stem cells, often implies overexpression of certain genes and other genetic manipulations. Besides, potential clinical application of MSCs may involve various differentiation procedures. While there are only a handful of works on the analysis of the influence of cryopreservation on gene expression in modified cells and cellular differentiation potential [26, 27], the influence of possible temperature fluctuations on these parameters remains unclear.

According to previously obtained data, temperature fluctuations in the steam storage device can range within –180 °C to –150 °C; rarely, in emergency situations, the temperature can rise to –130 °C. Moreover, the dry ice temperature of –78.5 °C or refrigeration equipment with a temperature of –80 °C are often used for transportation of biological samples or for temporary storage.

The aim of this study was to perform a comprehensive analysis and comparative characterization of possible changes in vital parameters and differentiation potential, as well as transgene expression of placental MSCs during temperature fluctuations within liquid nitrogen steam storage. Such an approach would mimic long-term preservation within the realities of practical biobanking, possible transportation on dry ice, and temporal storage in low temperature refrigeration equipment.

## Methods

### Experimental design

Placental amnion cells were isolated via application of a standard enzymatic method followed by characterization for MSC origin as described previously [5]. The obtained placental MSCs were subjected to differentiation by culturing in corresponding media as well as to genetic modification via transduction. Native and modified placental MSCs were frozen with a freezing protocol generally accepted for adherent stromal cell suspensions [22]. Part of the samples was subjected to repeated temperature fluctuations in the subzero range. The samples were comprehensively analyzed for characteristics of vital parameters, differentiation potential, and transgene expression after cryopreservation at a constant end temperature in comparison to cryopreservation with temperature fluctuations. All experiments were performed on the samples derived from at least three independent donors (*n* ≥ 3).

### Cell derivation and cell culture

#### Cell source, and ethical and legislative statement

Human placenta material was donated in an anonymized manner with written informed consent of the patients after routine Caesarian section at the Department of Gynaecology and Obstetrics at Hannover Medical School, Germany (approved by the Ethical Commission of Hannover Medical School, No. 2396-2014) and in Kharkiv municipal maternity hospital No.1, Ukraine (approved by Bioethics Committee of the Institute for Problems of Cryobiology and Cryomedicine of the National Academy of Sciences of Ukraine, No. 2-0306-2013). This study did not involve animal experiments.

#### Derivation of primary cell culture

Primary culture of MSCs was derived from the amnion of placenta via application of an enzymatic method [5]. Amnion membranes were washed in phosphate-buffered saline (PBS) supplemented with 10% ciprofloxacin (Fresenius Kabi, Bad Homburg, Germany) and then dissected into small pieces and incubated for 1 h at 37 °C in presence of 0.25% trypsin. After trypsin digestion the samples were filtered through a 100-μm cell strainer (BD Biosciences, Durhan, USA), followed by centrifugation of the obtained cell suspension for 5 min at 1200 rpm (Heraeus Multifuge 1S-R, Thermo Fisher Scientific GmbH, Dreieich, Germany). The supernatant was removed and the cell pellet was resuspended in MSC growth medium and plated on 10-cm cell culture dishes (Cellstar, Greiner BioOne, Frickenhausen, Germany).

#### Cell culture

Placental MSCs were cultured at 5% CO_2_ and 37 °C in a humidified CO_2_ incubator (Thermo Fisher Scientific GmbH, Dreieich, Germany) under sterile conditions in Dulbecco’s modified Eagle’s medium high glucose (DMEM LG; Lonza, Basel, Switzerland) supplemented with 1% (v/v) 100 mM sodium pyruvate solution (Lonza, Basel, Switzerland), 10% (v/v) fetal bovine serum (FBS; Lonza, Basel, Switzerland), 1% (v/v) antibiotic-antimycotic solution (BioWest, Nuaillé, France), and 1% (v/v) ascorbic acid (Sigma-Aldrich, St. Louis, USA) in 10-cm tissue culture plates (CELLSTAR®, Greiner BioOne, Frickenhausen, Germany). Harvesting of the cells was performed with application of 0.25% trypsin solution.

### Characterization of multipotent stromal cells

#### Reverse transcription polymerase chain reaction for MSC markers

Extraction of RNA was performed with the peqGOLD Total RNA Kit (Peqlab GmbH, Erlangen, Germany) according to the manufacturer’s protocol. In brief, the cell pellet was lysed in 400 μl RNA lysis buffer and transferred onto a DNA removing column for removal of contaminant DNA. Following centrifugation at 12,000 × g for 1 min, 400 μl of 70% ethanol was added to the flow through. The obtained lysate was loaded onto a Perfect Bind RNA Column and centrifuged at 10,000 – g for 1 min in order to bind RNA to the column. The column was washed once with 500 μl RNA Wash Buffer I and twice with 600 μl RNA Wash Buffer II. The column was then dried by centrifugation at 10,000 × g for 2 min. The total RNA was eluted from the column with 50 μl sterile RNase-free water. The concentration of RNA was measured with the NanoDrop photometer ND-1000 (Thermo Fisher Scientific GmbH, Dreieich, Germany). The RNA was transcribed to complementary DNA (cDNA) with application of a High Capacity cDNA Reverse Transcription Kit (Life Technologies GmbH, Darmstadt, Germany). The addition of Oligo (dT) primers (TIB Molbiol, Berlin, Germany) ensured that only the mRNA was transcribed.

Analysis of the mesenchymal markers was performed with the application of reverse transcription polymerase chain reaction (RT-PCR) set in 30 μl volume per sample as follows: 24 μl ddH_2_O (double distilled water), 3 μl 1× PCR buffer (NEB, Frankfurt, Germany), 1 μl 100 mM dNTPs (Fermentas, St. Leon-Rot, Germany), 0.5 units Taq Polymerase (NEB, Frankfurt, Germany), 0.5 μl of each primer (final concentration 0.3 μM), and 1 μg cDNA. The thermal cycling protocol included: a pre-cycling step at 95 °C for 3 min; 35 cycles of denaturation at 95 °C for 45 s; annealing at 60 °C for 45 s; extension at 72 °C for 90 s, and a final extension step at 72 °C for 10 min. Oligonucleotides were designed to have a 60 °C annealing temperature. A summary of the oligonucleotide sequences, product fragment sizes, and accession numbers is presented in Additional file 1.

#### Osteogenic, chondrogenic, and adipogenic differentiation

MSCs were differentiated into mesenchymal lineages by culturing in corresponding adipogenic, osteogenic, and chondrogenic differentiation media, as described previously [5].

Osteogenic differentiation was performed by culturing the cells under corresponding conditions. The cells were seeded at a 5 × 10^4^ cells/well concentration onto six-well culture plates (Cellstar, Greiner BioOne, Frickenhausen, Germany) followed by culturing for 21 days in DMEM LG (Biochrom AG, Berlin, Germany) supplemented with 20 mM HEPES zwitterionic buffer and 10% FCS HyClone™, 0.05 mM l-ascorbic acid-2-phosphate (Sigma-Aldrich, St. Louis, USA), 0.1 μM dexamethasone, 1% (v/v) penicillin/streptomycin and 3 mM sodium dihydrogen phosphate monohydrate (Carl Roth GmbH, Karlsruhe, Germany). Mineralization of cells differentiated into osteoblasts was detected by Von Kossa staining after 21 days. In brief, differentiated mineralized cells were washed two times with PBS and fixed with 10% formalin, then washed once with PBS and twice with ddH2O followed by the addition of 1% silver nitrate (Riedel de Haen GmbH, Germany). The cell culture dish was exposed to sunlight for 30 min, washed with ddH2O, and stained with 5% sodium thiosulfate (Sigma-Aldrich, St. Louis, USA) for 5 min, followed by a final rinse with ddH2O. Mineralization was visualized with a bright field Keyence Biozero microscope.

Chondrogenic differentiation was performed by pelleting 2.5 × 10^5^ cells/well by centrifugation for 5 min at 200 × g in v-shaped 96-well plates (Cellstar, Greiner BioOne, Frickenhausen, Germany) with further culturing in corresponding differentiation media for 21 days. The differentiation medium consisted of DMEM HG culture medium ((Biochrom AG, Berlin, Germany) supplemented with 20 mM HEPES buffer, 1% (v/v) penicillin/streptomycin, 1% (v/v) ITS Universal Cell Culture Supplement Premix (Becton Dickinson GmbH, Heidelberg, Germany), 0.1 μM dexamethasone, 0.17 mM l-ascorbin acid-2-phosphate (Sigma-Aldrich, St. Louis, USA), 0.35 mM l-proline (Biochrom AG, Berlin, Germany), 1 mM sodium pyruvate (Biochrom AG, Berlin, Germany), and 10 ng/ml transforming growth factor-β3 (PeproTech GmbH, Hamburg, Germany). The differentiation medium was changed every 3 days. After 21 days, the pellets were fixed with 10% paraformaldehyde (Carl Roth GmbH, Karlsruhe, Germany), cut in 7-μm sections and stained with Alcian blue (Sigma-Aldrich, St. Louis, USA) as an indicator of sulfated glycosaminoglycan (sGAG)-rich extracellular matrix.

For adipogenic differentiation the cells were seeded at a 5 × 10^4^ cells/well concentration onto six-well culture plates and cultured for 14 days in DMEM supplemented with 20% FCS HyClone™ (Thermo Fisher Scientific GmbH, Dreieich, Germany), 20 mM HEPES zwitterionic buffer (Biochrom AG, Berlin, Germany), 1 μM dexamethasone (Sigma-Aldrich, St. Louis, USA), 0.5 mM 3-isobutyl-1-methylxanthin (Sigma-Aldrich, St. Louis, USA), 60 μM indomethacin (Sigma-Aldrich, St. Louis, USA), 1% (v/v) penicillin/streptomycin (Biochrom AG, Berlin, Germany), and 10 μg/ml insulin (Sigma-Aldrich, St. Louis, USA). After 14 days the cells were stained with Oil Red O for visualization of lipid vacuole formation. In brief, the cells were washed with PBS, fixed for 20 min in 10% formalin (Sigma-Aldrich, St. Louis, USA), then rinsed twice with ddH_2_O and washed with 50% ethanol (AppliChem GmbH, Darmstadt, Germany). Lipid vacuoles were visualized with a bright field microscope (Keyence Biozero, Keyence, Osaka, Japan) after 10 min incubation in Oil Red O (Sigma-Aldrich, St. Louis, USA) followed by washing with acetone/50% ethanol (Merck KGaA, Darmstadt, Germany) and a final rinse with water.

Each differentiation procedure was performed in triplicate and compared with undifferentiated cells as controls (*n* = 3).

### Cryopreservation of cells

Placental MSCs were removed from culture plates, washed from trypsin with a culture medium, and equilibrated in cryoprotective medium at a concentration of 1 × 10^6^ cells/ml at 20 °C for 15 min. Culture medium supplemented with 10% DMSO (Sigma-Aldrich, St. Louis, USA) and 10% FBS was used as cryoprotective medium. The samples were placed in 1.8-ml cryovials (Nunc™ Thermo Fisher Scientific GmbH, Dreieich, Germany) and cooled in the programmable freezer “ЗП-10” (BioCold Special Designing and Technical Bureau, Kharkiv, Ukraine) with a cooling rate of 1 °C/min to –80 °C, followed by immersion into the liquid nitrogen within the storage devices (HB-05; JSC Ural Compressor Plant, Ekaterinburg, Russia) in standard cassette boxes. The samples were uniformly distributed in a box and surrounded on all sides by identical test tubes containing the medium. The samples were rapidly thawed in the water bath at +37 °C with subsequent washing of DMSO with a culture medium. Native cells from the cell culture were used as a positive control; cells resuspended in 96% ethanol at –18 °C and kept overnight with further washing with the culture medium were taken as the negative control.

To assess the impact of temperature fluctuations on placental MSCs, a method mimicking potential conditions of the practical biobanking was applied. In summary, the samples were removed from the cryostorage in standard cassette boxes and kept at +20 °C on a styrofoam platform to achieve the required temperature followed by immersion of the cassette with probes in cryovials back to the liquid nitrogen. The temperature in the cryovials was monitored with a dual-channel temperature controller (OWEN TRM 200; OWEN LLC, Moscow, Russia). Thermocouple sensors were immersed in the central position of identical cryovials with identical content. The software of the device manufacturer (OWEN Process Manager V. 1.2.) was applied for analysis.

The temperature in the samples was increased to –80 °C, –100 °C, and –150 °C. The number of temperature cycles was 5, 10, 20, 30, 40, and 50 for each temperature range.

### Evaluation of cell number, viability, and functional properties of the cells

The number of cells was counted in the Neubauer hemacytometer (Marienfeld-Superior, Lauda-Königshofen, Germany) by staining with methylene blue. The viability of cells was determined by staining with 0.4% trypan blue.

In order to detect and distinguish necrotic and apoptotic processes, which may have been initiated in the cells by cryopreservation procedures, the cells were analyzed with application of the APC Annexin V Apoptosis detection kit with PI (BioLegend, San Diego, USA) according to the manufacturer’s protocol. In brief, the cells were washed twice with cold BioLegend Cell Staining Buffer, and then resuspended in 100 μl Annexin V Binding Buffer. Then, 5 μl APC Annexin V and 10 μl propidium iodide (PI) solution were added. Cells were incubated for 15 min at room temperature (24 °C) in the dark. Then, 400 μl Annexin V Binding Buffer was added. Flow cytometry was performed with FACSCalibur™ (Becton Dickinson GmbH, Heidelberg, Germany). This kit is specifically designed for the identification of apoptotic and necrotic cells.

Adhesive properties were studied by seeding 2 × 10^5^ cells per well on a six-well plate, and culturing at 5% CO_2_ and 37 °C in a humidified CO_2_ incubator. After 24 h incubation nonattached cells were aspirated and the remaining adhered cells were detached with 0.25% trypsin and counted.

The MTT test was used to study metabolic and proliferation activity of the cells. CellTiter 96® Non-Radioactive Cell Proliferation Assay (Promega, Fitchburg, USA) was applied according to the manufacturer’s protocol. In brief, 1 × 10^4^ cells per well were seeded with four parallels for each sample on a 96-well plate (Cellstar, Greiner BioOne, Frickenhausen, Germany) and cultivated for 24 h in 100 μl MSC medium in a humidified CO_2_ incubator at 37 °C and 5% CO_2_. After incubation for 24 h, 15 μl MTT reagent per well was added with incubation for 4 h at 37 °C. Then, 100 μl Stop Mix reagent was added per well and incubated further for 1 h at room temperature in the absence of light. Finally, the formazan concentration was measured with a BioRad-680 microplate reader (BioRad, Munich, Germany) at the 570 nm wavelength.

The resazurin reduction test was also used for the analysis of metabolic activity of the cells in order to support the MTT data. This was performed by plating the cells at a concentration of 1 × 10^5^ cells/ml per well in 1 ml of culture medium onto 24-well plates (SPL Life Science, Pocheon-Si, South Korea) followed by the addition of 200 μl resazurin solution (Sigma-Aldrich, St. Louis, USA) dissolved in PBS at a concentration of 0.15 mg/ml. The mixture was incubated for 24 h in a humidified CO_2_ incubator under standard conditions. The absorption was measured with a Solar PV 1251C spectrophotometer (Solar, Minsk, Belarus) at a wavelength of 570 nm.

### Cryomicroscopy

Cryomicroscopic studies were performed for visualization of changes during cryopreservation, temperature fluctuations, and thawing. Placental MSCs were removed from the culture plates using 0.25% trypsin-EDTA, washed with PBS, and resuspended in cryopreservation medium. After 15 min of pre-incubation in cryopreservation medium, 50 μl of the cell suspension was collected from the bottom of the cryotube and pipetted in a cryomicroscopic chamber complex (KP 6; SDTB IPC&C, Kharkov, Ukraine) on the base of a microscope (MBI 15U 4.2; LOMO, St. Petersburg, Russia). The samples were covered with glass slide covers to prevent drying. Freezing was carried out at 1 °C/min down to –100 °C, followed by raising of the temperature to –80 °C at a rate of 1 °C/min and lowering back to –100 °C, with this cycling being repeated three times. The temperature range was selected with respect to the most significant changes in the cell vital parameters which were identified in our research and to the capacity of available cryomicroscopic equipment. Sample was thawed at the rate of 1 °C/min until complete melting of the ice crystals. The sizes of the individual crystals, channels between the crystals, cells, and cell aggregates were recorded with a digital camera (UCMOS 3100; SIGETA, Hangzhou, China) and analyzed with Toup View v.3.7. (Hangzhou ToupTek Photonics Co. Ltd, Hangzhou, China) and ImageJ v.1.48 r (NIH, Public domain) software. Since the sizes of crystals and channels varied widely, the measurement and analysis was performed on the same elements at different temperatures.

### Microarray analysis

mRNA from the samples was purified with the QIAGEN RNeasy RNA Isolation Kit (QIAGEN, Hilden, Germany) according to the manufacturer’s protocol. For minimization of technical variations, each RNA sample was generated in duplicate and pooled prior to labeling and hybridizing.

The Microarray utilized in this study represents a refined version of the whole human genome oligo microarray 4x44K v2 (Design ID 026652; Agilent Technologies, Santa Clara, USA) called ‘054261On1M’ (Design ID 066335) developed at the Research Core Unit Transcriptomics (RCUT) of Hannover Medical School. Microarray design was created at Agilent’s eArray portal using a 1 × 1 M design format for mRNA expression as a template. All noncontrol probes of design ID 026652 have been printed five times within a region comprising a total of 181,560 features (170 columns × 1068 rows). Four of these regions were placed within one 1 M region giving rise to four microarray fields per slide to be hybridized individually (customer specified feature layout). Control probes required for proper feature extraction software operation were determined and placed automatically by eArray using recommended default settings.

Synthesis of Cy3- or Cy5-labeled cRNA was performed with the Low Input Quick Amp Labeling kit, two color (#5190-2944; Agilent Technologies, Santa Clara, USA) according to the manufacturer’s recommendations.

cRNA fragmentation, hybridization, and washing steps were carried out as recommended in the two-color microarray-based gene expression analysis protocol V6.7, except that 1000 ng of each fluorescently labeled cRNA population was used for hybridization.

Slides were scanned on the Agilent Micro Array Scanner G2565CA (pixel resolution 3 μm, bit depth 20). Data extraction was performed with the feature extraction software V10.7.3.1 using the extraction protocol file GE2_107_Sep09.xml, except that the ‘multiplicative detrending’ algorithm was inactivated.

Measurements of on-chip replicates (quintuplicates) were averaged using the geometric mean of processed intensity values of the green channel (‘gProcessedSignal’; (gPS and of the red channel (‘rProcessedSignal’; rPS) to retrieve one resulting value per channel and unique noncontrol probe. Single features were excluded from averaging if they: i) were manually flagged; ii) were identified as outliers by the feature extraction software; iii) lay outside the interval of 1.42 × interquartile range regarding the normalized gPS distribution of the respective on-chip replicate population; or iv) showed a coefficient of variation of pixel intensities per feature that exceeded 0.5.

Averaged PS values were normalized by the quantile normalization approach.

Finally, a lower intensity threshold (surrogate value) was defined based on the intensity distribution of negative control features. This value was fixed at 5 normalized PS units. All measurements below this intensity cutoff were substituted by the respective surrogate value of 5.

Gene regulation was calculated for the samples differentiated after cryopreservation procedures with constant temperatures and temperature fluctuations in comparison to native cell differentiation.

### Transduction of the cells with various constructs

Lentiviral transduction of the cells was performed according to standard procedures, as described previously [28]. In brief, 3.5 × 10^5^ cells were seeded onto six-well plates 24 h before transduction and cultivated at 37 °C with 5% CO_2_. After 24 h the culture medium was replaced with fresh MSC medium supplemented with protaminsulfate (8 μg/ml) and the cells were incubated for 30 min prior to transduction at 37 °C with 5% CO_2_. Next, the lentivirus-containing supernatant with MOI 1 (multiplicity of infection) was added to the medium. Two different constructs were used for transduction. Transduction efficiency was evaluated after 3 days by measuring the percentage of green fluorescent protein (GFP)-positive cells with the FACSCalibur™ (Becton Dickinson GmbH, Heidelberg, Germany) flow cytometer with the rate of 10,000 events per measurement. Analysis of cytofluorimetric data was carried out with the Flowing Software V. 2.1 program.

Construct 1: Lentiviral expression vector construct pRRL.PPT.T11.EGFP.hPGK.M2.pre (kindly provided by Michael Morgan, MHH, Hannover, Germany) contained GFP as a reporter gene. Such a construct represents a chemically inducible system. After transduction, 48 h of induction with 1 μg/ml doxycycline (Sigma-Aldrich, St. Louis, USA) gives a maximum expression level, which returns to background levels after removing the induction reagent (doxycycline) in approximately 5 days.

Construct 2: Lentiviral expression vector pLVTHM contained GFP as a reporter gene. Lentiviral particles were produced with application of the DNA-PEI method by transfecting 6 × 10^6^ of 293 T cells (DSMZ, Braunschweig, Germany) with 5 μg psPAX2 encoding gag and pol proteins, and with 5 μg pMD2G containing VSV-G protein.

Transduction was performed in three independent samples for each construct prior to cryopreservation procedures and compared with the same noncryopreserved samples as controls.

### Data analysis and statistics

The data was collected from three to five independent experiments in triplicate (*n* ≥ 3). Statistically significant conclusions were obtained by analysis of mean values and standard deviation calculations (mean ± SD) with Mann-Whitney criteria and Fisher’s method. Parameter changes were considered statistically significant at *p* < 0.05. Statistical calculations and data analysis were performed with the application of Statgraphics V 2.1 software (Statpoint Technologies, Inc., Warrenton, USA).

## Results

### Characterization of cells for MSC origin

**Fig. 1:**
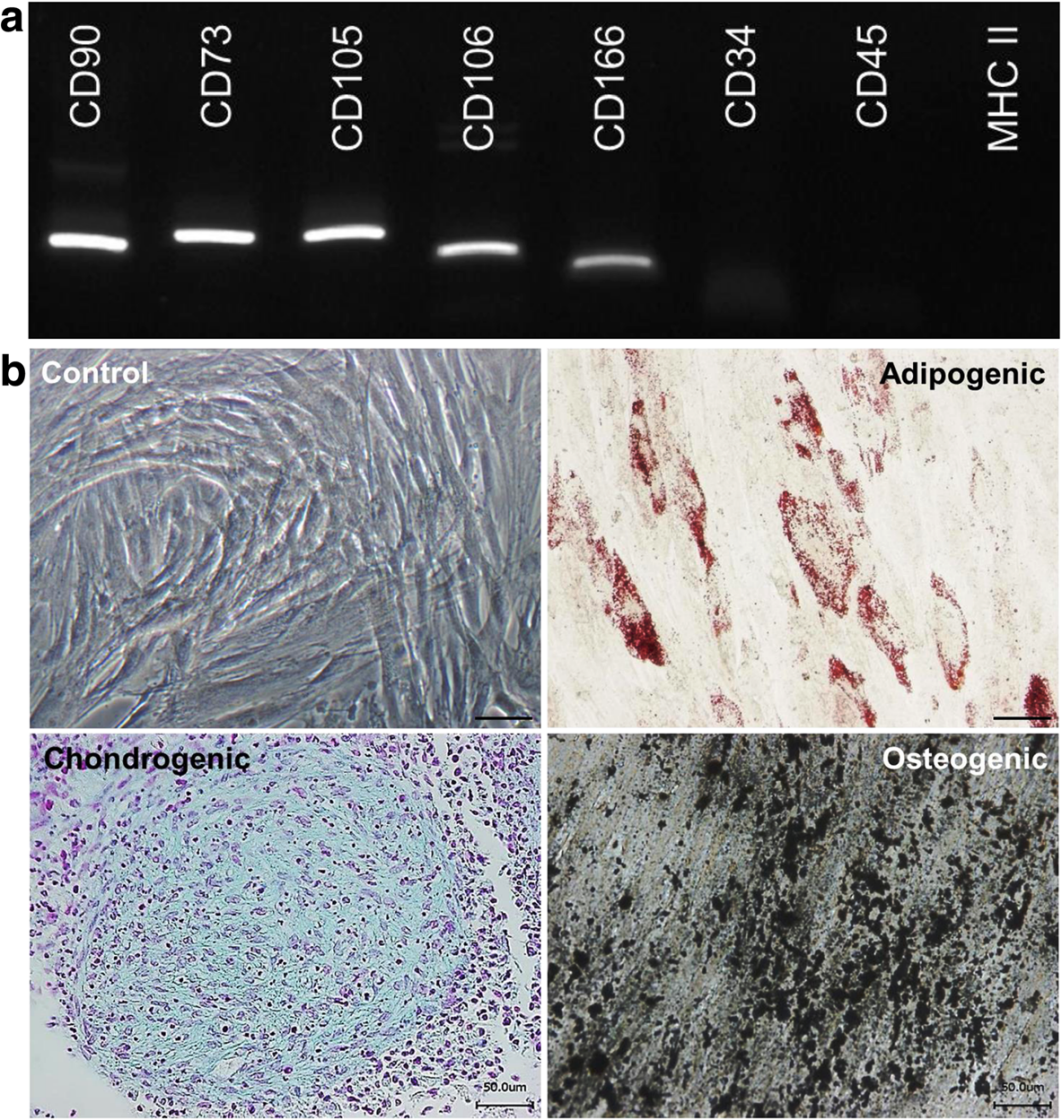
Characterization of primary cell culture derived from placenta for MSC origin. **a** RT-PCR analysis for expression of typical MSC markers: cells provide a signal for CD90, CD73, CD105, CD106, and CD166 genes while being negative for CD34, CD45, and MHC class II. **b** Differentiation potential of primary cell culture derived from placenta into lineages of MSC origin: adipogenic (Oil Red O staining); chondrogenic (Alcian blue staining); and osteogenic (Von Kossa staining). Cells expanded in standard MSC culture medium were considered as a negative control. *Scale bars* = 50 μm

Primary cells obtained from placental amnion displayed typical fibroblast-like spindle-shaped morphology in cell culture conditions, were adherent to plastic, and capable of forming a monolayer. RT-PCR analysis revealed that cells were positive for typical MSC markers, such as CD90, CD73, CD105, CD106, and CD166, and negative for the hematopoietic markers CD34 and CD45 as well as negative for MHC class II (Fig. 1a). In our previous studies we also confirmed the expression of CD73 and CD105 markers in these cells at the protein level with flow cytometry analysis [5, 18]. Furthermore, the cells were capable of differentiating into adipogenic, chondrogenic, and osteogenic mesenchymal lineages in corresponding culture conditions (Fig. 1b). Such features are considered to be sufficient for characterization of a cell line as MSCs [29].

### Analysis of the influence of temperature fluctuations during low temperature storage on the state of placental MSCs

#### Thermograms of temperature fluctuations

**Fig. 2:**
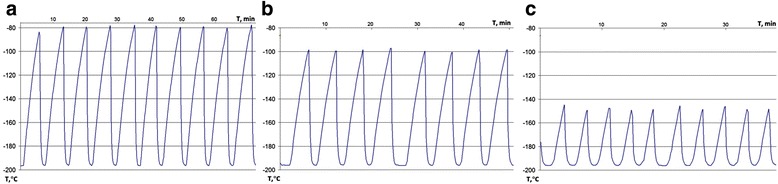
Thermograms of temperature fluctuations within the studied samples. Dynamics of temperature changes in multiple cycles of the temperature ranges: **a** between –196 °C and –80 °C; **b** between –196 °C and –100 °C; **c** between –196 °C and –150 °C

To evaluate the actual temperature fluctuations which may occur in the studied samples during long-term preservation, we analyzed the thermograms of temperature changes directly in the samples contained in cryotubes within standard storage cassette boxes (Fig. 2). Dynamics of temperature changes in multiple cycles were studied in the temperature ranges between –196 °C and –150 °C, –196 °C and –100 °C, and –196 °C and –80 °C (Fig. 2). Samples from the lower end temperature in the liquid nitrogen (–196 °C) were freely thawed at room temperature (20 °C) to the higher end temperatures of the cycling range (–150 °C, –100 °C, and –80 °C), followed by immersion into liquid nitrogen to return to the lower end temperature of the cycle.

#### Cryomicroscopy

**Fig. 3:**
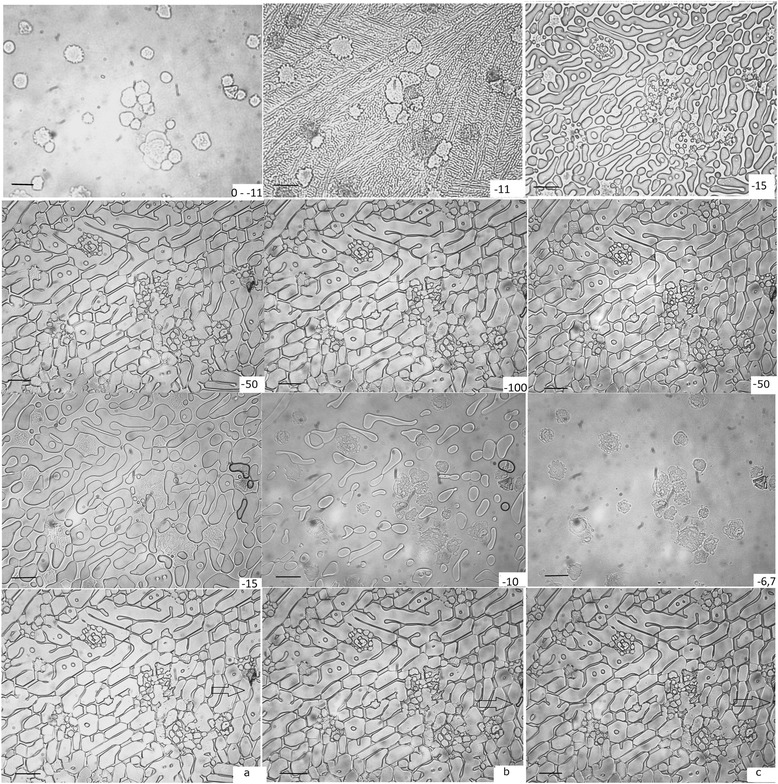
Cryomicroscopic analysis of crystal formation during temperature fluctuations. Freezing of placental MSCs from 0 °C to –100 °C and thawing from –100 °C to –6.7 °C from *left* to *right*, *top* to *bottom* (the temperature is indicated on each image). **a** Samples at –80 °C during the first cycle of temperature fluctuations; **b** samples at –100 °C during the last cycle of temperature fluctuations; **c** samples at –80 °C during the last cycle of temperature fluctuations. *Arrows* indicate the change of inclusions. *Scale bars* = 20 μm

The cell concentration at the bottom of the cryovials after 15 min was around 6 × 10^6^ cells/ml, while the initial concentration was 1 × 10^6^ cells/ml, which is explained by sedimentation of cells at the bottom. Lowering the temperature down to –100 °C at a rate of 1 °C/min resulted in rapid crystal formation at –11 °C with generation of a multitude of small 1.4 ± 0.12 μm crystals with channels in between. The crystal growth was observed with decreasing temperature, resulting in the fusion of individual crystals and narrowing of the channels between the crystals (Fig. 3). The crystals acquired a pointed shape and separate crystals fused together.

Two types of crystals have been identified: larger crystals in the intercellular space and small crystals located around the cells. The formation of small crystals, in our opinion, is related to aspirates of the cell membrane or the release of water from the cell. The crystals acquire their final shape and dimensions at –50 °C to –60 °C. Visually, further cooling did not result in changes in the crystals. At a temperature of –100 °C large crystals (11.8 ± 2.4 μm in width and 33.0 ± 11.2 μm in length) were observed in the intercellular space, and small crystals (5.6 ± 1.6 μm in diameter) were located around the cells.

During warming of the sample, the start of crystal melting was visually observed at –60 °C, with expansion of the individual channels and receding of individual crystals from each other. Gas bubbles between the crystals enlarged (indicated by arrows in the figure). At a temperature of –6.9 °C melting of the crystals terminated and the gas bubbles were fully dissolved.

Cyclical fluctuations in the temperature range between –80 °C and –100 °C did not result in significant changes in cell size, size of the cell aggregates, crystals, and channels. Changes in the transparency of the crystals were observed which may be due to equipment characteristics. An increase in the size of gas bubbles was observed during lowering of the temperature, which may be associated with a decrease in the ice volume (Fig. 3).

#### Viability, state, and functional parameters of the cells

**Fig. 4:**
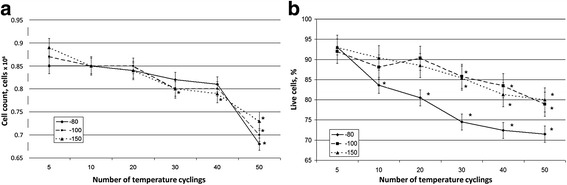
Cell count and cell survival after cryopreservation procedures. **a** The number of cells in the studied samples, depending on the number of temperature fluctuations. **b** Cell survival after temperature fluctuations by trypan blue staining. *p < 0.05, versus samples with no temperature fluctuations

Analysis of cell number after frequent temperature fluctuations in the samples shows that a decrease depended on the number of temperature cycles and did not depend on the range of temperature variations. A statistically significant decrease was observed only after 30–40 cycles of temperature fluctuations (Fig. 4a).

Analysis with trypan blue staining demonstrated a decrease in the cell survival depending on the number of cycles as well as on the temperature range. Cell survival at temperature fluctuations down to –80 °C decreases most drastically, whereas the temperature fluctuations to –100 °C and –150 °C are not significantly different to each other (Fig. 4b.).

Flow cytometry with Anexin V and PI, which allows us to distinguish necrotic and apoptotic events, indicates that cell viability after temperature fluctuations decreases significantly, and to a greater degree at fluctuations to –80 °C than to –150 °C or –100 °C, with significance reached after 10 cycles of temperature fluctuation. Apoptotic changes in cells are minor, but more pronounced with temperature fluctuations to –80 °C (Table 1).

**Table 1:** Flow cytometry with Annexin V and PI for analysis of necrotic and apoptotic processes in the cells after the studied cryopreservation procedures (mean ± SD)

		FACS Anexin V with PI			
		Anexin V–,PI– (no necrosis or apoptosis), %	Anexin V–,PI + (necrosis), %	Anexin V+,PI– (apoptosis), %	Anexin V+,PI + (necrosis and apoptosis), %
Constant cryopreservation temperature, –196 °C		83.88 ± 3.7	1.02 ± 0.05	1.33 ± 0.89	13.7 ± 1.7
Temperature fluctuations between –196 °C and –80 °C	5 cycles	82.7 ± 2.1	1.19 ± 0.03	2.21 ± 0.8*	14.66 ± 2.0
	10 cycles	68.2 ± 2.1*	1.12 ± 0.02	2.1 ± 0.09*	27.98 ± 2.2*
	20 cycles	56.8 ± 1.5*	0.54 ± 0.01	2.5 ± 0.08*	41.47 ± 3.82*
	30 cycles	54.2 ± 3.1*	1.01 ± 0.02	3.05 ± 0.09*	42.68 ± 2.09*
	40 cycles	52.5 ± 1.8*	0.72 ± 0.02	2.65 ± 0.15*	43.64 ± 1.28*
	50 cycles	51.8 ± 1.9*	0.86 ± 0.01	1.83 ± 0.05*	46.22 ± 2.38*
Temperature fluctuations between –196 °C and –100 °C	5 cycles	81.6 ± 2.8	0.48 ± 0.02	1.67 ± 0.06	16.09 ± 1.44
	10 cycles	80.2 ± 2.9	0.58 ± 0.02	2.17 ± 0.07*	17.47 ± 1.88*
	20 cycles	72.0 ± 2.5*	0.89 ± 0.01	2.03 ± 0.02*	26.05 ± 3.54*
	30 cycles	70.6 ± 3.1*	1.18 ± 0.02	2.96 ± 0.05*	24.11 ± 4.37*
	40 cycles	64.8 ± 2.4*	0.65 ± 0.04	3.23 ± 0.01*	25.96 ± 2.78*
	50 cycles	59.6 ± 3.3*	1.6 ± 0.01	1.15 ± 0.01	38.94 ± 3.64*
Temperature fluctuations between –196 °C and –150 °C	5 cycles	88.37 ± 2.7	0.55 ± 0.01	1.94 ± 0.01	11.82 ± 3.02
	10 cycles	83.6 ± 1.8	0.83 ± 0.07	1.8 ± 0.09	13.64 ± 1.1
	20 cycles	80.02 ± 3.2	0.57 ± 0.06	1.59 ± 0.03	17.68 ± 2.81
	30 cycles	78.79 ± 4.5*	0.34 ± 0.02	1.57 ± 0.03	19.30 ± 0.77*
	40 cycles	70.39 ± 4.1*	0.98 ± 0.02	1.3 ± 0.03	25.46 ± 3.39*
	50 cycles	64.49 ± 3.6*	0.96 ± 0.08	1.6 ± 0.05	27.74 ± 2.7*

*p < 0.05, versus constant cryopreservation temperature control

PI propidium iodide

**Fig. 5:**
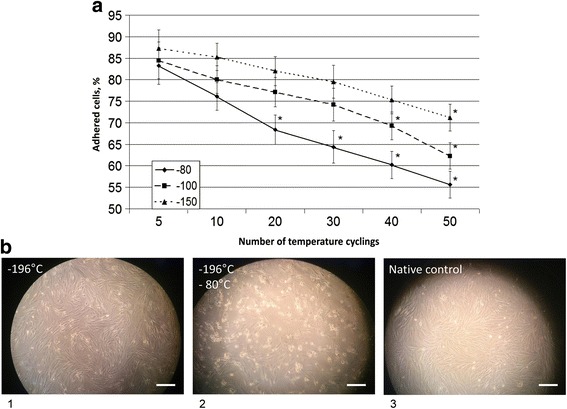
Adhesive properties of the cells 24 h after cryopreservation with constant temperatures and temperature fluctuations. **a** Number of cells capable of adhering to plastic 24 h after thawing. **b** Typical example of cellular adherence after studied cryopreservation procedures: *1* samples stored at constant temperatures; *2* samples after 50 cycles in the range of temperature fluctuations between –196 °C and –80 °C; *3* native control without cryopreservation. *Scale bars* = 100 μm

The ability of the cells to adhere to plastic 24 h after thawing was performed as a functional test. It was shown that the adhesive properties of the cells were significantly compromised after temperature fluctuations during cryopreservation (Fig. 5). Counting the cells that adhered 24 h after thawing revealed a drop similar to the tendency for cell survival (Fig. 5a). Figure 5b demonstrates a typical visual example of the cells 24 h after seeding: the cells after 50 cycles in the highest range of temperature fluctuations (–196 °C to –80 °C) display highly compromised adhesive properties (Fig. 5b2) in comparison to samples stored at constant temperatures (Fig. 5b1), or to the native control without cryopreservation (Fig. 5b3). Multiple floating nonadhered cells can be seen (Fig. 5b2).

**Fig. 6:**
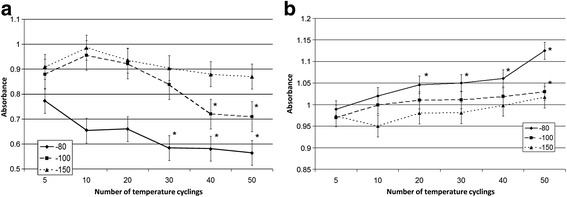
Metabolic and proliferation activity of the cells after cryopreservation procedures. **a** MTT test for placental MSCs after temperature fluctuations. **b** Resazurin reduction test for placental MSCs after temperature fluctuations. *p < 0.05, versus samples with no temperature fluctuations

Analysis of cellular parameters with the MTT test demonstrated that the metabolic activity of the cells after thawing depends both on the range of temperature fluctuation and on the number of fluctuations. MTT reduction was highest at temperature fluctuations down to –80 °C (Fig. 6a). At the same time, metabolic activity did not significantly decrease with temperature fluctuations down to –100 °C and –150 °C, at least within 30 cycles.

Resazurin reduction also allows evaluation of the mitochondrial metabolic activity in the cells [30]. Metabolic activity measured with reduction of resazurin after temperature fluctuations dropped in a manner similar to the activity of the MTT test (Fig. 6b). There were no significant changes until 20 cycles of temperature fluctuations in any of the studied temperature ranges; significant changes occur after 40–50 cycles in the fluctuation range up to –80 °C.

#### Differentiation potential of placental MSCs after temperature fluctuations during low temperature storage

**Fig. 7:**
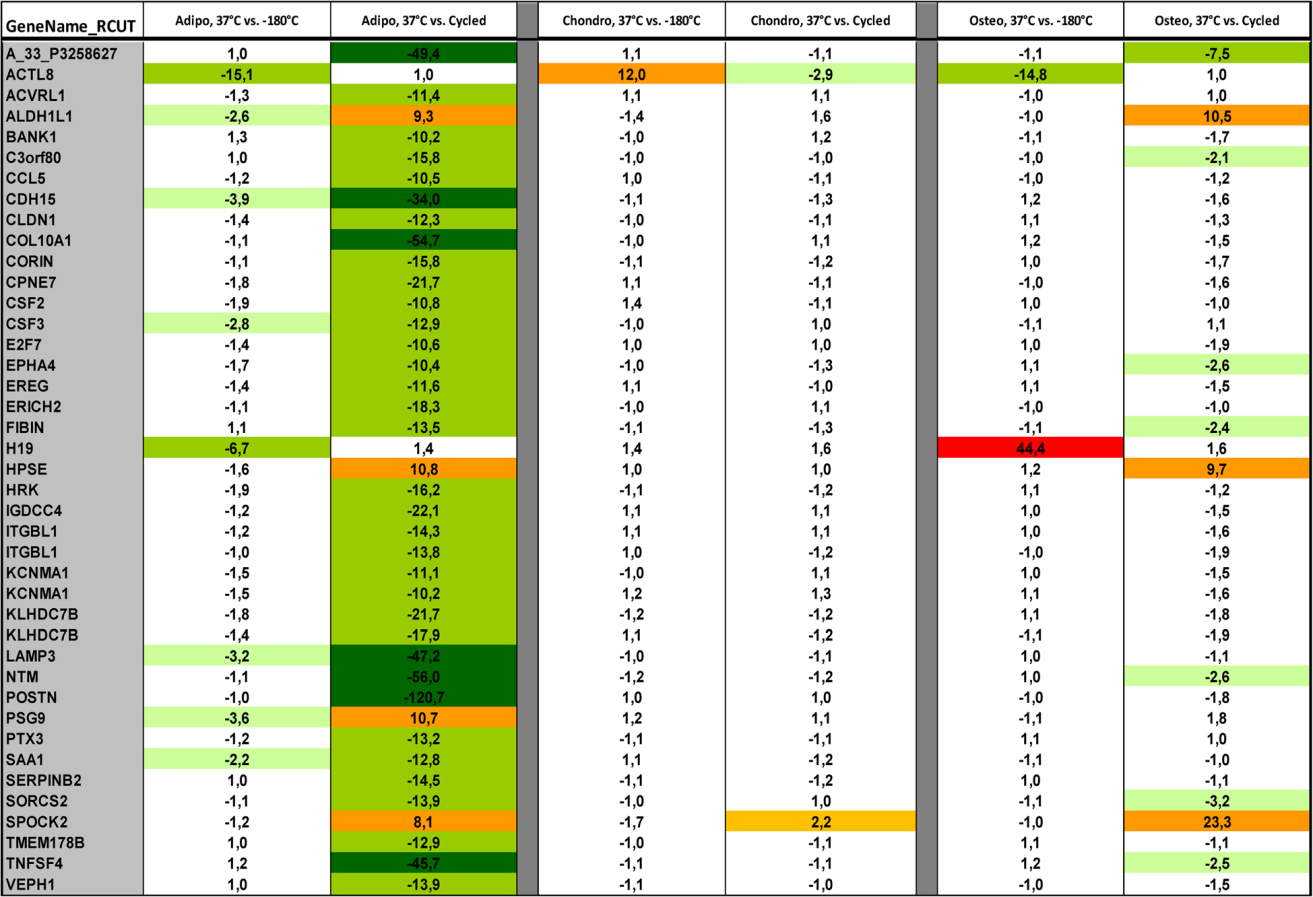
Microarray analysis of gene regulation after cryopreservation procedures. Gene regulation in the samples differentiated after cryopreservation procedures with constant temperatures and temperature fluctuations in comparison to the native cell differentiation. Genes with over tenfold regulation. *Adipo* adipogenic, *Chondro* chondrogenic, *Osteo* osteogenic

Analyzed samples were able to differentiate into adipogenic, chondrogenic, and osteogenic lineages after cryopreservation with temperature fluctuations that mimic long-term storage, visually identically to the samples stored at constant temperature as well as to the native noncryopreserved control, also used in the MSC characterization (Fig. 1). However, microarray analysis of 34,127 genes revealed that nearly 0.38% of all studied genes in adipogenic, 0.24% in chondrogenic, and 0.35% in osteogenic lineages were either upregulated or downregulated more than twofold and up to tenfold in the samples differentiated without cryopreservation procedures in comparison to the samples differentiated after cryopreservation at the constant temperatures (Additional file 2). Interestingly, the gene-specific regulation pattern was very different in the samples differentiated after cryopreservation with thermal cycles (Additional file 2). In percentage values, 3.08% of genes in adipogenic, 0.35% in chondrogenic, and 0.95% in osteogenic lineages were either upregulated or downregulated more than twofold and up to tenfold in comparison to the samples differentiated without cryopreservation procedures (Additional file 2). Moreover, 41 genes were regulated more than 10-fold in the samples differentiated after cryopreservation procedures in comparison to native cell differentiation (Fig. 7). In future studies, quantitative differentiation assays after temperature fluctuations should be evaluated for better understanding of the impact on such processes.

#### Analysis of the influence of temperature fluctuations during low temperature storage on transgene expression in placental MSCs

**Fig. 8:**
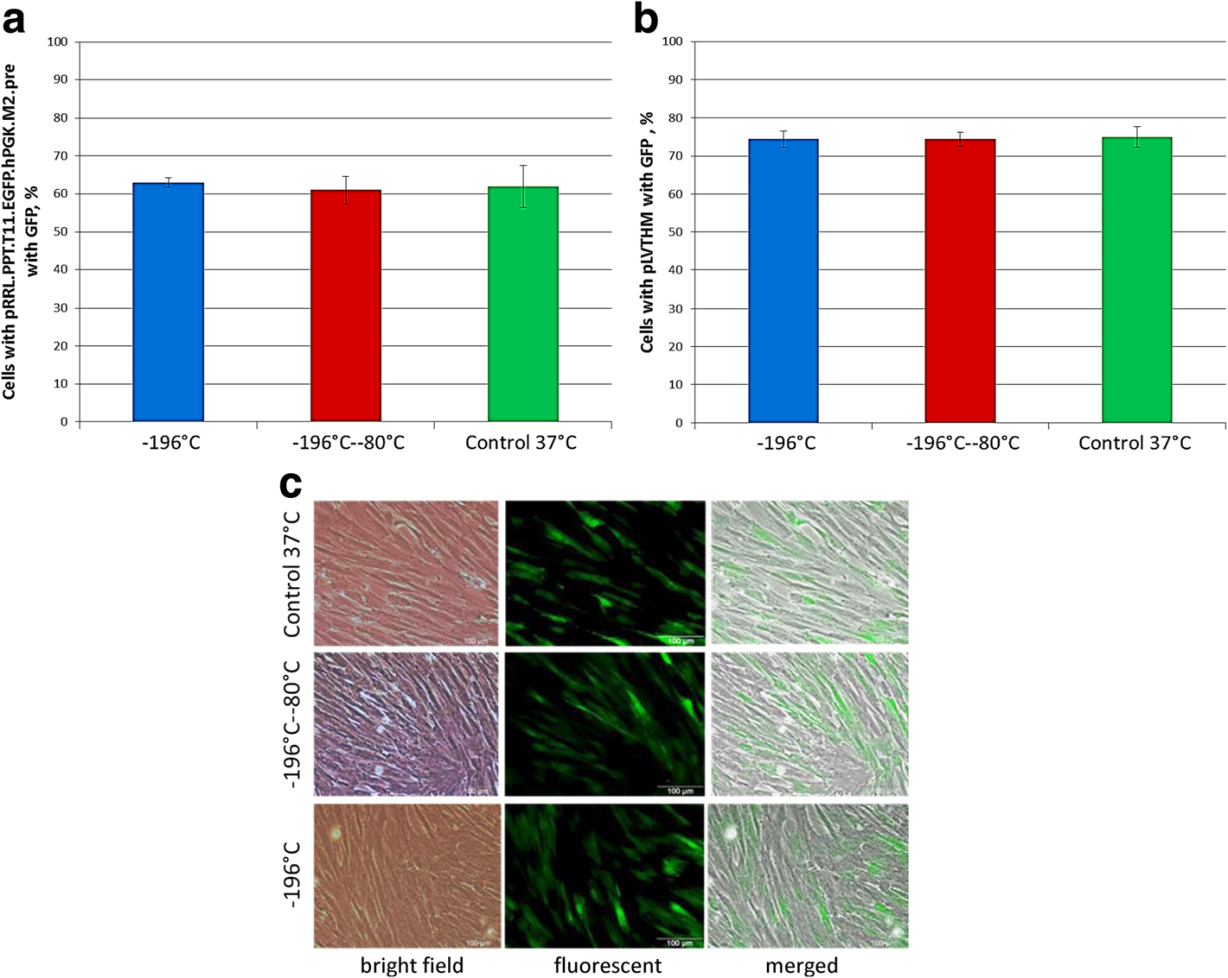
Influence of temperature fluctuations on transgene expression in placental MSCs. Cells with expressed transgene after 24-h recovery from the studied cryopreservation conditions: **a** lentiviral expression vector construct pRRL.PPT.T11.EGFP.hPGK.M2.pre with green fluorescent protein (*GFP*) as a reporter gene; **b** lentiviral expression vector pLVTHM with GFP as a reporter gene; **c** typical example of expression of transgenic vector with GFP reporter

Cryopreservation with 50 cycles of temperature fluctuations between –196 °C and –80 °C was chosen to evaluate the influence on transgene expression in placental MSCs as the condition with the most pronounced effect on the other studied parameters. Two independent groups of studied cells were transduced with different constructs with GFP as a reporter gene. The samples were evaluated 24 h after recovery from cryopreservation conditions, or 24 h after repassaging in the case of noncryopreserved control. Neither cryopreservation under constant temperature conditions nor temperature fluctuations during cryopreservation have a significant impact on the expression of transgene constructs in our sample groups (Fig. 8). Therefore, no concerns about transgene expression emerge during long-term cryopreservation of placental MSCs.

## Discussion

The use of real low-temperature banks for biological objects is often accompanied by certain temperature fluctuations associated with a variety of handling events, manipulations of the stored samples, transportation, etc. An accumulation of occurrence of such events in the long term may result in alterations within the samples which may compromise the outcome of cryopreserved material [23–25]. This is especially important for patient-specific material or for stocks of frozen samples intended for use in regenerative medicine which can be stored for decades prior to actual application. Placental MSCs are among the most highly prolific as well as already practically applied cell types which fall into this category [7–9, 31].

In this study we aimed to analyze a wide range of parameters of placental MSCs with regard to the effect of mimicked long-term cryopreservation accompanied by temperature fluctuations in the samples. We developed a model that allows modeling of the oscillations of temperature inherent to the probable events in a practical biobank. The lower end temperature in our experiments was –196 °C (liquid nitrogen storage temperature) and the upper end temperatures were –150 °C, –100 °C, and –80 °C. The upper end temperatures mimicked the range found in available mechanical cryogenic freezers, dry ice, and liquid nitrogen steam storage, which could contribute to temperature fluctuations during practical long-term biobanking.

The cryomicroscopic studies involved a range of temperatures which additionally included thawing points. Cycles of temperature fluctuations were performed only in the –100 °C to –80 °C range due to limitations of equipment parameters. Cryomicroscopy revealed that melting of the ice crystals and expansion of the channels in between them starts at temperatures above –60 °C to –50 °C. Changes associated with melting could be observed at temperatures above –40 °C to –30 °C, which is consistent with our previous studies [18] showing that a sufficient cell survival rate requires at least –40 °C end temperature during slow freezing. Temperature fluctuations within –80 °C to –100 °C resulted in minor changes related to ice volume alterations. Additional studies are necessary for a deeper understanding of the role of temperature fluctuations on cell damage caused by crystal formation.

Vital parameters, which characterize structural and functional conditions of the cells after thawing, have been used to analyze the influence of temperature fluctuations during cryopreservation. The majority of survival rates provided in cryopreservation protocols are based on trypan blue staining data, which mainly indicate the membrane integrity and efficiency of efflux pumps [32]. However, early necrotic and apoptotic events in the cells cannot be distinguished by such an approach, while vital parameters might already be irreversibly compromised by cryodamage. Therefore, in addition to the classic Coulter Counter method of trypan blue staining, we evaluated the cells with the flow cytometric Annexin V apoptosis detection kit with PI, which allowed us to differentiate between the contribution of necrosis and apoptosis on cell survival [33]. Additionally, the ability of the cells to adhere to plastic after post-thawing recovery was performed as a functional test. We observed a significant decrease in the adhesive properties of the cells stored with temperature fluctuations that mimic long-term biobanking. This also corresponds with the observations of other authors who analyzed the actual storage of MSCs for over 20 years [34]. Such an effect may be associated with the observed necrotic and apoptotic events, triggered in the cells by repeated thermal cycles, as well as with the utilization of a large amount of metabolic sources for recovery processes after the stress accumulated during cryopreservation [35].

Analysis of the metabolic condition of the studied cells after thawing showed similar patterns in MTT and resazurin reduction tests. Certain variances between the results of the MTT test and resazurin reduction test may be explained by methodological peculiarities connected with a different incubation period of reagent components (being 4 hours and 24 h, respectively).

The data analysis allows us to conclude that temperature fluctuations within –196 °C to –150 °C result in significant changes in the quantity, viability, and functional activity of placental MSCs after 20 cycles; apoptotic changes in this case are not observed. Significant changes at more than 20 cycles are also observed after temperature fluctuations within –196 °C to –100 °C. However, the quantity, viability, and functional activity of placental MSC do not differ significantly from those parameters after temperature fluctuations within –196 °C to –150 °C.

Interestingly, impact of the temperature fluctuations between –196 °C and –80 °C on the quantity, viability, and metabolic activity of placental MSCs is significantly different in comparison to constant temperature, and is more pronounced than in the –196 °C to –150 °C and –196 °C to –100 °C ranges.

Numerous research groups have demonstrated the capability of placental MSCs to differentiate after cryopreservation [27, 36]. A study of the parameters of bone marrow MSCs after 20 years of cryopreservation was able to differentiate them into osteoblasts, adipocytes, and neuronal cells [34]. We were also able to differentiate the cells after cryopreservation with temperature fluctuations into three mesenchymal lineages, which were visually identical to the noncryopreserved control and to the samples with constant cryopreservation temperature conditions. However, microarray analysis revealed patterns of gene regulation that were different between the samples cryopreserved at constant temperatures and those with temperature fluctuations. Such gene regulation did not visually affect the outcome of the differentiation procedures in any of the three differentiation lineages; however, this effect requires further analysis. Interestingly, gene regulation of differentiated cells after cryopreservation with thermal cycling was highly expressed in the adipogenic lineage: the number of genes with minor regulations (from twofold up to tenfold) was almost an order of magnitude higher in comparison to noncycled samples, while 37 genes were regulated more than 10-fold and in one case more than 100-fold in comparison to native cell differentiation. Such an effect should be considered and studied in cases of adipogenic differentiation after long-term cryopreservation, as well as analysis of the functional characteristics of the highly upregulated genes.

Since a wide range of genome manipulations is applied these days in biomedical research, we also aimed to analyze the influence of temperature fluctuations in our model on the levels of expression of transgene vectors. Other researchers demonstrate that cryopreservation does not affect expression of a transgene [26]. This was also confirmed in our study.

In summary, a developed model mimicking possible events in long-term practical biobanking by repeated temperature fluctuations in cryopreserved samples allowed us to rapidly evaluate changes in placental MSCs. Certain alterations in comparison with material stored at constant temperature were observed. Such a model can be considered in the studies of long-term preservation of other biological objects which would help develop further strategies and evaluate potential limitations.

## Conclusions

Significant structural changes in the ice crystals and channels were detected during our cryomicroscopy study in the temperature range above –40 °C. At temperature fluctuations below –80 °C, slight changes in the inclusion and the transparency of the crystals were observed.

The total number of cells, viability, and metabolic and apoptotic parameters of placental MSCs did not significantly differ from controls with temperature fluctuations in the range of –196 °C to –100 °C in less than 20 cycles. Increasing the number of cycles, as well as the temperature fluctuation range to –80 °C, leads to significant lowering of cell number, viability, and metabolic characteristics after thawing. The number of apoptotic changes increases depending on the number of cycles of temperature fluctuation.

The adhesive properties of MSCs after thawing are significantly compromised in the cells that were cryopreserved with temperature fluctuations in comparison to cryopreservation with the constant end temperatures.

Short-term temperature fluctuations to –100 °C, possible during practical storage of placental MSCs in low-temperature biobanks, do not significantly affect the cellular characteristics. Temperature fluctuations up to –80 °C lead to compromise of cell culture properties.

The possibility of placental MSCs differentiating into mesenchymal lineages is not compromised after cryopreservation with a constant end temperatures as well as after cryopreservation with temperature fluctuations in comparison to native cells. However, regulation of various genes after cryopreservation procedures significantly varies in differentiated cells.

Transgene expression is not compromised after cryopreservation with a constant end temperature as well as after cryopreservation with the studied range of temperature fluctuations in comparison to native cells.

Therefore, temperature fluctuations in the samples, typically inherent to practical biobanking, should be considered during long-term cryostorage of placental MSCs. While the differentiation potential of these cells is not significantly compromised, alterations in vital parameters and gene regulation are observed. This should be especially considered for any future clinical application of placental MSCs in the field of regenerative medicine. The development of strategies for avoiding such temperature fluctuations is feasible and should not be overlooked.

## Additional files

Additional file 1: Oligonucleotides with corresponding accession numbers. (DOC 32 kb)
Additional file 2: Microarray analysis. Gene regulation in the samples differentiated after cryopreservation procedures with constant temperatures and temperature fluctuations in comparison to the native cell differentiation. Genes with regulation from twofold to 10-fold. (XLSX 382 kb)

## Abbreviations

GFP: Green fluorescent protein
GMP: Good Manufacturing Practice
MSC: Multipotent stromal cells
