# Supplementary material for: Influence of temperature fluctuations during cryopreservation on vital parameters, differentiation potential, and transgene expression of placental multipotent stromal cells
Source: Stem Cell Res Ther. 2017 Mar 11;8:66. doi: 10.1186/s13287-017-0512-7 (PMC5346212; doi:10.1186/s13287-017-0512-7)
Supplement: Additional file 1: — Oligonucleotides with corresponding accession numbers. (DOC 32 kb) [file 13287_2017_512_MOESM1_ESM.doc]

| **Gene** | **Sequence** | **Fragment size (bp)** | **Accession number** |
| --- | --- | --- | --- |
| **CD 90** | 5´- AGC AAG GAC GAG GGC ACC TA - 3´  5´- AGG TGT TCT GAG CCA GCA GG- 3´ | 136 | NM_001311162.1 |
| **CD 73** | 5´- ATG GCT CCT CTC AAT CAT GC - 3´  5´- ATC AAT GGG CGA CCG GAT AC - 3´ | 158 | NM_001204813.1 |
| **CD 105** | 5´- GCC GAC GAC GCC ATG ACC CT - 3´  5´- GCA TGC CAC AGC TGG AGT AA - 3´ | 154 | NM_001114753.2 |
| **CD 106** | 5´- TGG ATT CTG TGC CCA CAG TAA - 3´  5´- TGG TCA CAG AGC CAC CTT CT - 3´ | 120 | NM_001199834.1 |
| **CD 166** | 5´- ACG TGT TTG AGG CAC CTA CAA - 3´  5´- AGC TGC TCT GTT TCG AGA AAC A - 3´ | 94 | NM_001243281.1 |
| **CD 34** | 5´- CGC TTT GCT TGC TGA GTT TG - 3´  5´- TGA GAC ACA GGG TGC AGG CT – 3 | 175 | XM_011510170.1 |
| **CD 45** | 5´- TTC CTG CAG AAC CCA AGG AAT - 3´  5´- TCC ATC CCT GCA GTG AAT GAG - 3 | 128 | XM_006711474.2 |
| **MHC II** | 5´- CTG ACT CCC AAC AGA GCG CC - 3´  5´ - GCC TGA TTG GTC AGG ATT CA - 3 | 165 | NM_019111.4 |
